# An Approach to Nasopharyngeal Mass in Newborns: Case Series and Systematic Literature Review

**DOI:** 10.5041/RMMJ.10463

**Published:** 2022-01-27

**Authors:** Roee Noy, Liron Borenstein-Levin, Arie Gordin

**Affiliations:** 1 Department of Otolaryngology—Head and Neck Surgery, Rambam Health Care Campus, Haifa, Israel; 2 The Ruth & Bruce Rappaport Faculty of Medicine, Technion–Israel Institute of Technology, Haifa, Israel; 3 Neonatal Intensive Care Unit, Ruth Rappaport Children’s Hospital, Rambam Health Care Campus, Haifa, Israel

**Keywords:** Congenital nasopharyngeal mass, glioma, heterotopic brain tissue, teratoma

## Abstract

**Objective:**

Congenital nasopharyngeal masses (CNMs) are rare. Presenting symptoms vary, and the differential diagnoses cover a wide spectrum of possibilities. As it is uncommon, most examples discussed in literature are described as case reports or series. Guidelines on CNM patient management do not exist. In this study, we present two (2) cases of neonates with CNMs that were encountered at our tertiary center. Additionally, to best elaborate a comprehensive, case-based approach to CNM management, we offer an up-to-date, diagnosis-to-treatment review of current literature.

**Methods:**

Case series and systematic literature review.

**Results:**

Twenty-eight (28) studies are included since January 2000 to October 2021, with a total of 41 cases. Most common diagnosis was teratoma (78%). Female-to-male ratio was 2.5:1. Twenty percent of cases presented prenatally with polyhydramnios or elevated alpha-fetoprotein. Postnatally, the presenting symptoms most frequently encountered were respiratory distress (78%), oral mass (52%), and feeding difficulties (29%). Seventy-five percent of affected newborns showed symptoms within the first 24 hours of life. Forty percent of cases had comorbidities, especially in the head and neck region.

**Conclusions:**

Congenital nasopharyngeal masses can be detected antenatally, or symptomatically immediately after birth. Airway protection is a cornerstone in the management. Selecting the right imaging modality and convening a multidisciplinary team meeting are important toward the planning of next steps/therapeutic approach. Typically, a transnasal or transoral surgical approach will be deemed sufficient to address the problem, with a good overall prognosis.

## INTRODUCTION

The nasopharynx—the embryonic intersection of the neural axis and alimentary and respiratory tracts—is subject to a variety of congenital anomalies. Congenital head and neck irregularities are rare, with a 5.5% prevalence among all congenital anomalies.^1^ Congenital nasopharyngeal masses (CNMs) can be divided into two categories, such as benign neoplasm versus malignant neoplasm (Table 1). 2

**Table 1:** Differential Diagnosis, Congenital Nasopharyngeal Mass (CNM).

Type of Neoplasm	Differential Diagnosis
**Benign Neoplasm**	Adenoid tissue or retention cyst*
	Aneurysmal bone cyst
	Angiofibroma
	Antrochoanal polyp*
	Ectopic pituitary
	Fibrous dysplasia
	First branchial pouch cyst*
	Giant cell tumor
	Hairy polyp*
	Hemangioma
	Heterotopic brain tissue (e.g. cephalocele, glioma)
	Mucocele*
	Pleomorphic adenoma
	Pyogenic granuloma*
	Salivary hamartoma
	Sinonasal polyp*
	Teratoma (can be malignant)
	Thornwaldt cyst*
**Malignant Neoplasm**	Carcinoma
	Esthesioneuroblastoma
	Hematological malignancies (e.g. lymphoma, leukemia)
	Metastases (e.g. neuroblastoma)
	Rhabdomyosarcoma

* Cystic/Polypoid benign neoplasm

As opposed to anterior nasal masses, which are usually seen on external examination of the nose, posterior nasal cavity and nasopharyngeal masses are often difficult to visualize. Moreover, CNM diagnosis can be delayed since signs and symptoms are seldom specific and may mimic other upper respiratory tract problems. However, while feeding difficulties may be the only presenting symptom in patients with smaller lesions, larger CNMs usually present with acute respiratory distress and may require emergent airway protection. Therefore, as newborns are obligate nasal breathers, it is essential to quickly diagnose and treat situations with nasopharyngeal obstruction.

Due to the rarity of cases, most CNMs described in the literature are case reports or series. This study presents two cases treated in our tertiary center, along with a systematic review of the current literature. Our purpose is to offer an up-to-date, case-based approach to CNM management, from diagnosis to treatment.

## CASE PRESENTATION #1

**Figure 1:**
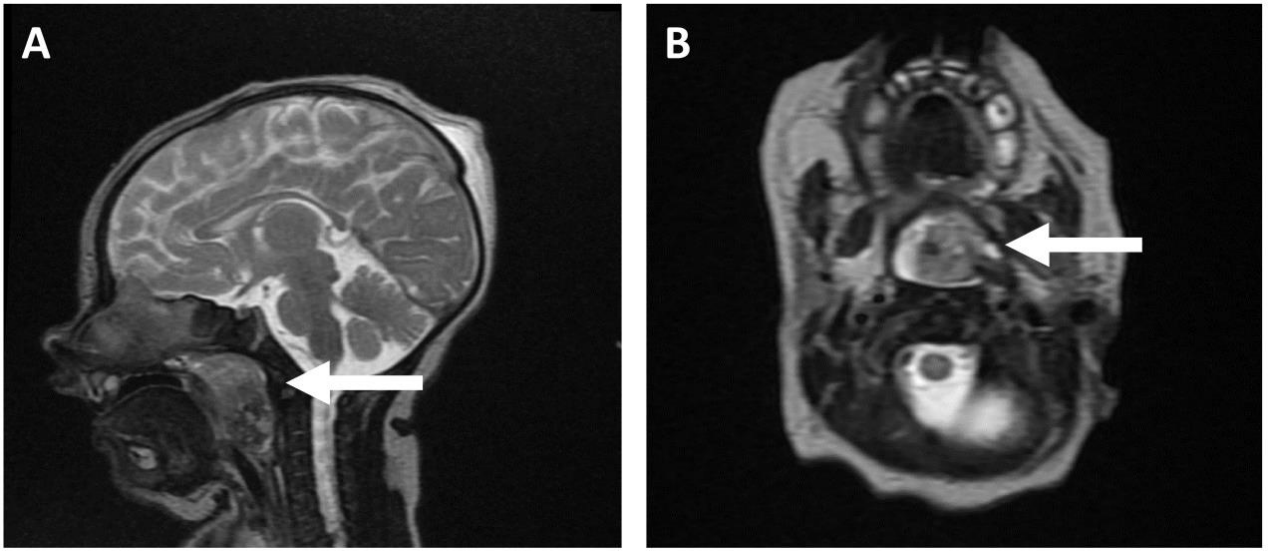
MRI with a Nasopharyngeal Mass Bulging into the Oropharynx and the Base of Tongue, with No Intra-cranial Involvement. **A:** The lesion consists of restricted areas, areas with decreased susceptibility-weighted imaging (SWI) values. **B:** Posteriorly, there is a 0.5 cm area with a high T1-weighted signal, and a medium-to-low signal on fat-suppression.

A 3400-g female neonate was born to a primigravida, healthy mother, by a vaginal delivery, at 40+1 weeks’ gestation. Delivery was uneventful, with 9/10 Apgar scores and normal physical exam. Prenatal evaluation revealed polyhydramnios, which was attributed to gestational diabetes. During the first hours of life, dyspnea, desaturation events during feeding, and increased salivation were noticed. A nasogastric tube passed with difficulty through both nostrils; therefore an anatomical blockage was suspected. Nasal endoscopy was performed and revealed a nasopharyngeal mass. Protective intubation was performed, and the neonate was then transferred to our neonatal intensive care unit (NICU). Magnetic resonance imaging (MRI) revealed a heterogeneous lesion bulging from the nasopharynx into the oropharynx and the base of tongue, which resembled radiographically a teratoma (Figure 1). A biopsy, performed under general anesthesia, suggested the presence of glial tissues, a component of teratoma. Three days later, a transoral surgical coblation procedure was carried out. The completion of the resection included a transnasal part, which resulted in a fully macroscopic excision and patent airway. The neonate was extubated on postoperative day 1, with no respiratory symptoms or feeding problems, and was discharged from the hospital on postoperative day 3. The final histopathological report revealed a mature teratoma. Follow-up one month after surgery was unremarkable, with no evidence to suggest recurrence.

## CASE PRESENTATION #2

**Figure 2:**
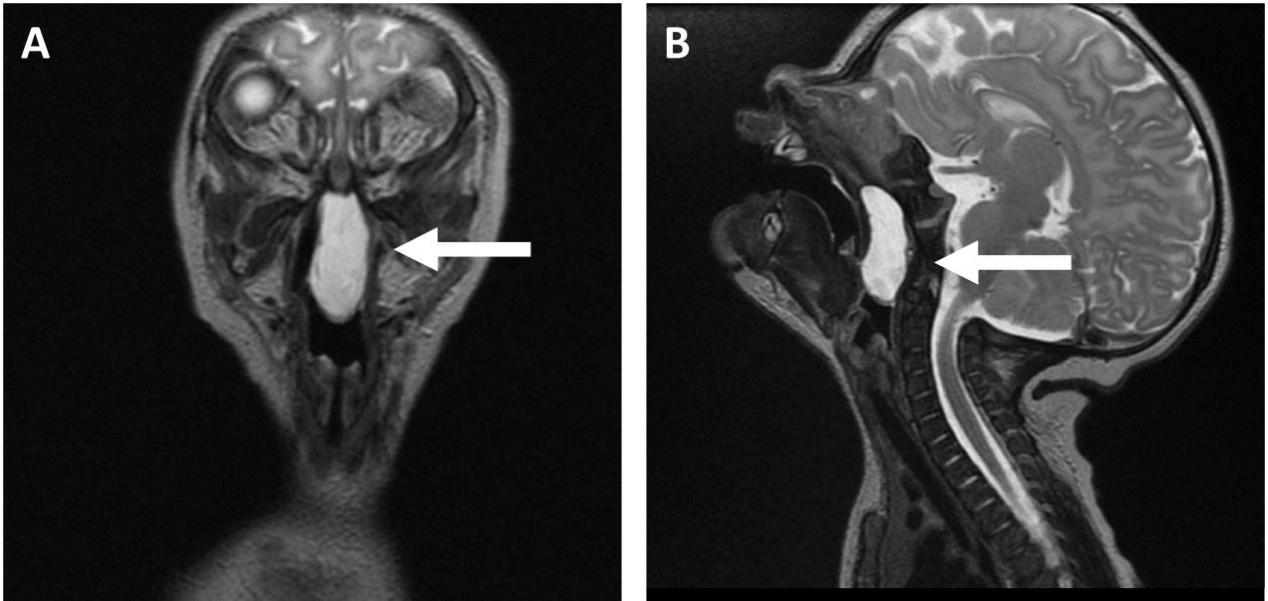
MRI with a Midline Nasopharyngeal Cystic-elliptical Mass, with Well-defined Borders and No Intra-cranial Involvement. **A:** Lesion begins in the posterior aspect of the nose and reaches the oropharynx. **B:** Sagittal plane.

Two days old, a male neonate presented with feeding and breathing difficulties. Physical examination was normal except for mild dyspnea and micro-retrognathia. Desaturation and dyspnea were relieved when the neonate was placed prone after insertion of a nasal airway. History revealed an uneventful pregnancy and spontaneous vaginal delivery at 38+6 weeks’ gestation, with a birth weight of 3785 g. Prenatal evaluation was normal. Laboratory studies and echocardiography were normal, and the neonate was transferred to our NICU for further evaluation for upper airway obstruction. Upon arrival, a nasal endoscopy exam was performed, revealing a cystic mass blocking the nasopharynx and the soft palate. A neck ultrasound (US) demonstrated a midline cystic mass posterior to the tongue. Brain US was unremarkable. Head and neck MRI demonstrated an elliptical, cystic, well-defined mass with fluid content and a stipe with partition extending from the posterior aspect of the nose to the oropharynx (Figure 2).

As well-defined planes were observed between the mass and its adjutant structures, no biopsy was taken preoperatively. Transnasal endoscopic surgery was carried out to fully resect macroscopically a cystic nasopharyngeal mass. Extending from the roof of the nasopharynx to the soft palate, the mass, which presented with some liquid and liquefactive properties during surgery, was entirely blocking passage to the choana. Due to epistaxis, postoperative care required the administration of one blood unit and a local tamponade with tranexamic acid. The neonate was extubated on postoperative day 2. Mild stridor was treated with budesonide inhalation. On postoperative day 7, the neonate was discharged home with no feeding or respiratory symptoms.

Later, on histopathological exam, the tumor was found to be positive for glial fibrillary acidic protein, suggesting the presence of brain, and the diagnosis of heterotopic brain tissue, which is similar to nasal glioma, was confirmed. Twelve months after surgery, the child was examined in our follow-up clinic for noisy breathing and snoring. Nasal endoscopy and MRI showed nasopharyngeal midline tissue, which was suspicious for enlarged adenoid tissue. Endoscopic excision of the tissue was performed, during which time no other suspicious nasopharyngeal mass was observed. Pathology report confirmed that the tissue consisted of epithelium and fibrotic stroma, which was consistent with adenoid hypertrophy.

## LITERATURE REVIEW

The goal of our systematic review was to find and discuss congenital nasopharyngeal masses (CNMs), as this topic is not well described in literature, and no management guidelines exist.

### Methods

**Figure 3:**
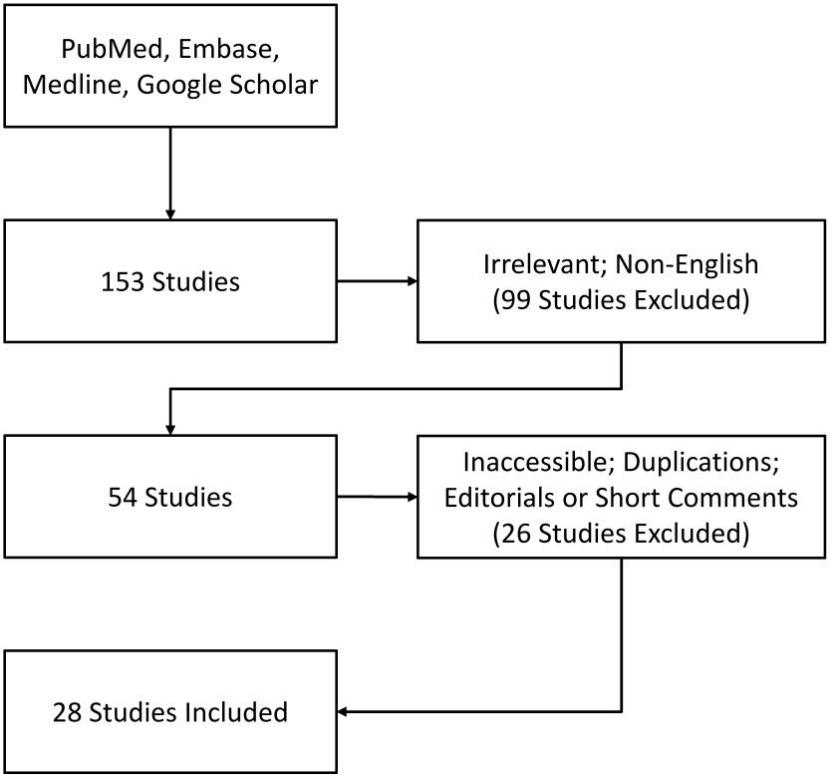
Systematic Literature Review Methodology.

According to the Preferred Reporting Items for Systematic Reviews and Meta-Analyses (PRISMA) statement, we searched PubMed, Embase, MEDLINE, and Google Scholar from January 2000 to October 2021 using the following search terms: “congenital nasopharyngeal mass” (or tumor or lesion), or “teratoma,” “heterotopic neuroglial tissue,” “brain tissue,” or “cephalocele.” From this, we identified 153 citations and fully reviewed reports (Figure 3). After excluding duplications, alternative diagnoses, and articles not in English, we had 22 case reports and 6 case series, for a total of 28 studies (41 cases) for inclusion in our review (Table 2). One reviewer screened each report retrieved. In each report we collected number of cases, gender, age, presenting symptoms, management, need for airway protection, usage of antibiotics or additional treatments, final histopathology report, and long-term follow-up or prognosis. Risk of bias in each study was relatively small as studies only discuss their own case reports or series, and no statistical analysis was required.

**Table 2:** Case Reports and Series of Congenital Nasopharyngeal Masses (CNMs).

Authors	Year	Country	Cases	Sex	Age	Symptoms	Pathology	Comments
Coppit et al.^3^	2000	USA	3	M, F	Birth	RD, FD, OM	Dermoid, teratoma	Prenatal alpha-fetoprotein and ultrasound, recurrence after 5 months
Behar et al.^4^	2001	USA	1	M	Birth	RD	Heterotopic neuroglial tissue	Tracheostomy
Uchino et al.^5^	2001	Japan	1	F	Birth	OM	Teratoma	Syndrome, dysmorphism
Andronikou et al.^6^	2003	Australia	3		Birth	RD	Teratoma	Polyhydramnios, prenatal MRI, EXIT procedure
Roh^7^	2004	Korea	1	F	7 mo	Snoring, sleep apnea	Hairy polyp	Adjacent to eustachian tube
Abdulkader et al.^8^	2006	Qatar	1	F	3 mo	RD	Hairy polyp	Comorbidity
Freitas et al.^9^	2007	Brazil	2	M, F	Birth	RD, OM	Teratoma	
Hossein & Mohammad^10^	2007	Iran	1	F	Birth	OM	Teratoma	Prenatal alpha-fetoprotein, comorbidity
Maartens et al.^11^	2009	Netherlands	1	M	Preterm	RD, nasal mass	Teratoma	Prenatal alpha-fetoprotein, polyhydramnios, intubation
Turnbull et al.^12^	2009	UK	1	F	Preterm	OM	Teratoma	Prenatal ultrasound, comorbidity
Tiwari et al.^13^	2009	India	1	F	Birth	RD, cyanosis	Teratoma	
He et al.^14^	2010	China	2	M, F	Birth	RD, FD, OM	Teratoma	Comorbidity
Mirshemirani et al.^15^	2011	Iran	1	F	Birth	FD, OM	Teratoma	Comorbidity
Chariker et al.^16^	2011	USA	1	F	3 d	FD	Teratoma	Comorbidities, pituitary duplication
Rangachari et al.^17^	2012	India	1	M	5 d	RD	Teratoma	Multiple intubations
Koike et al.^18^	2013	Japan	1	F	3 mo	RD	Hairy polyp	Respiratory failure
Bayır et al.^19^	2014	Turkey	1	M	Birth	RD	Teratoma	Polyhydramnios, multiple biopsies
Han et al.^20^	2014	Korea	1	M	Preterm	RD, FD, OM	Teratoma	Comorbidities, 2-step surgery
Mann et al.^21^	2014	UK	3	F	Birth	RD, FD, OM	Choristoma	
Radhakrishnan et al.^22^	2015	India	1	F	Birth		Salivary gland anlage tumor	Prenatal MRI, EXIT procedure
Menezes & Simao^23^	2015	Brazil	1		Preterm	RD, OM	Teratoma	Prenatal ultrasound, polyhydramnios
Hwang et al.^24^	2015	Australia	1	F	Birth	RD, OM	Teratoma	Polyhydramnios, tracheostomy, coblation, biopsy with wrong diagnosis
Alexander et al.^25^	2015	UK	6	M, F	Birth	RD, FD, OM	Teratoma	Hyponasal speech, intubation
Ghatage et al.^26^	2016	India	1	F	3 d	RD, FD	Teratoma	Comorbidities, tracheostomy
Jadhav et al.^27^	2017	India	1	F	Birth	RD, OM	Teratoma	
Thong et al.^28^	2018	Malaysia	1	F	24 h	RD, OM	Teratoma	Prenatal alpha-fetoprotein, comorbidities, delayed surgery
Aramesh et al.^29^	2020	Iran	1	F	Birth	RD, OM	Teratoma	Polyhydramnios
Kobayashi et al.^30^	2020	Japan	1	M	Birth	RD	Teratoma	Prenatal alpha-fetoprotein, maxillectomy due to recurrence

d, days; EXIT, *ex-utero* intrapartum treatment; F, female; FD, feeding difficulties; h, hours; M, male; mo, months; MRI, magnetic resonance imaging; OM, oral mass; RD, respiratory distress; US, ultrasound.

### Results

The most common diagnoses for CNMs were teratomas (78%, 32 patients) followed by hairy polyp (7%, 3 patients). Twenty percent of cases presented prenatally with either polyhydramnios or elevated alpha-fetoprotein levels. Female-to-male ratio was 2.5:1. Biopsies were performed in 25% of cases.

The most common presenting symptoms were respiratory distress (78%), oral mass (52%), and feeding difficulties (29%). There was a combination of symptoms in 33%.

Seventy-five percent of patients showed symptoms within the first 24 hours of life, 10% within the first month, and the rest within the first year.

Imaging was used in 72% of cases (30 patients)—of these, 55% had a computed tomography (CT) scan, and 45% had MRI. In 20% of cases, both modalities were used.

Forty-one percent of cases (17 patients) had co-morbidities, especially in the head and neck region: of them 60% had cleft palate, 30% were found to have systemic diseases (Pierre Robin sequence, West syndrome), and about 10% of patients had anatomical abnormalities (e.g. micrognathia). Fifty percent of cases occurred in developing countries.

## DISCUSSION

The nasopharynx represents the most superior portion of the pharynx, bounded superiorly by the skull base and inferiorly by the soft palate, and is lined by stratified squamous epithelium and by respiratory epithelium at the roof and nasal choanae. The nasopharynx derives from the neural crests of the ectodermal leaflet.^2^ As it is the embryonic intersection of the neural axis and the alimentary and respiratory tracts, the nasopharynx is subjected to a variety of congenital anomalies.

In the literature CNMs are rare, and most research is based on case reports and series. The purpose of this study was to review relevant cases in a select body of literature and to develop a diagnostic approach to CNMs, regardless of the histopathological type of the mass.

**Figure 4:**
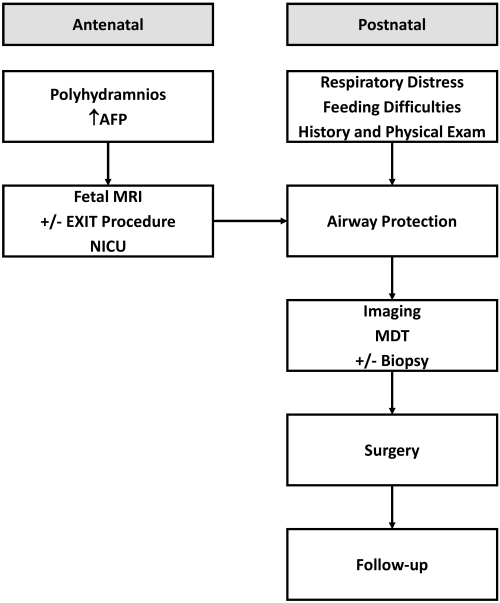
Schematic Approach to a Congenital Nasopharyngeal Mass (CNM) in a Newborn. AFP, alpha-fetoprotein; EXIT, *ex-utero* intrapartum treatment; MDT, multidisciplinary team; MRI, magnetic resonance imaging; NICU, neonatal intensive care unit.

We presented two cases of newborns who were admitted to our hospital with CNMs leading to airway obstruction. Both cases were managed similarly, although the final histopathological reports revealed different tumor types. In our literature review, most CNM cases were teratomas, but other tumors were described as well. Based on the cases we presented and our systematic literature review, we suggest the following approach to CNM management (Figure 4).

Prenatally, CNMs may present with polyhydramnios due to impaired swallowing.^6^,^11^,^19^,^23^,^24^,^29^ Elevated maternal alpha-fetoprotein levels may also be present.^10^,^11^,^17^,^30^ Ultrasound may detect these lesions.^12^,^23^ When a suspicion is raised from an ultrasound, a fetal MRI is highly recommended.^6^,^22^

Any lesion, especially in the head and neck area, requires referral to a genetic consultation. Parents should be referred to a tertiary hospital with an adequate team of anesthesiologists, neonatologists, and neonatal intensive care unit that can be prepared to carry out a potentially complicated airway management. Prenatal diagnosis also enables planning for an *ex-utero* intrapartum treatment (EXIT) procedure to secure a newborn’s airway before division of the maternal–fetal circulation in cases of large lesions.^6^,^22^ However, in most cases the EXIT procedure is probably unnecessary. Potential risks include anesthetic challenges and postpartum hemorrhage. The fact that most of the analyzed cases occurred in developing countries raises a concern regarding prenatal diagnostic processes in those countries.

The most common presenting symptoms for CNMs in these infants were respiratory distress, oral mass, and feeding difficulties. Airway protection is, therefore, a cornerstone in the management of CNMs. In a newborn who is suspected of upper airway obstruction, a blocking mass should be suspected. Otolaryngology consultation is advised, and preventive intubation should be considered. In the case of a difficult intubation, placement of a tracheostomy tube is advisable.

Although no such cases were described in the literature, it should be mentioned that CNMs can block the eustachian tube and potentially cause serous otitis media.

Prior to delivery of a child with CNM, it is highly advisable to convene a multidisciplinary team (MDT) consisting of neonatologists, otolaryngologists (head and neck surgeons), radiologists, and anesthesiologists. Following the birth, the group should meet again and discuss the next steps.

Selecting the appropriate imaging modality is mandatory. The purpose of imaging is to better acquaint physicians with the components of a given lesion and its relation to adjacent structures, especially the dura mater and the brain. Although an MRI with contrast agent is the recommended modality—as it easily distinguishes different types of tissues and offers reduced radiation exposure in comparison to a CT—its limited availability, long duration, and the need for patient sedation are disadvantages. A CT with contrast agent is an alternative, but its execution may require sedation, as well, and is associated with increased lifetime risk for cancer mortality (as follow-up imaging tests may be necessary). Based on our literature review, we conclude that it is not advisable to perform any CNM-related surgery without imaging, as it will increase the chances for incomplete resection, major bleeding, or adverse outcomes. When there is a concern regarding synchronic malformations, neonatologists may also require additional imaging tests.

A presurgical biopsy can inform physicians about the characteristics of a given lesion and its level of aggressiveness, as well as guide their decision-making regarding the extent of resection necessary, the need for additional surgeries, follow-up period intervals, and the need for adjuvant therapies. Due to the fear of bleeding or cerebrospinal fluid leak, it is not recommended to biopsy a vascularized lesion or one with a direct connection to the dura (thus reinforcing the need for imaging before proceeding to a final diagnosis and surgical remedies). As only 25% of cases in the analyzed literature were biopsied before surgery, the question may be raised whether a presurgical biopsy is necessary at all. Knowing the characteristics and aggressiveness of the mass can dictate the extent and the importance of macroscopically negative margin. Regardless, sometimes the urgent need for surgery can overcome the usefulness of carrying out a biopsy.

Fiberoptic intubation is the preferred technique for establishing airway access in patients with suspected masses that bulge to the oropharynx; nasal intubation is not advised as the nasopharyngeal mass may be injured, resulting in bleeding. Surgeons must be prepared with an option of surgical airway (tracheostomy) in case of failed intubation, especially in patients with large lesions. Tracheostomy techniques in newborns require experienced teams and is beyond the scope of our article.

Depending on the extent of a given mass, several surgical approaches are accepted. Most cases in the studied literature were handled via the transnasal approach or combined with the transoral approach. However, recurrent or aggressive tumors may require a more definite approach or even open surgery. Once again, airway management requires a multidisciplinary approach (anesthesiologists, neonatalogists, and otolaryngologists).

Parents of newborns must be included in the process of decision-making and choosing treatment options.

If preoperative imaging suggests an isolated mass with no relation to brain or dura mater, there is a low risk for meningitis or other major infectious sequelae. Based on systematic literature review, postoperative prophylactic antibiotic was not indicated, unless symptoms of infection (ill-appearing child, fever, neutrophilic leukocytosis, elevated C-reactive protein or erythrocyte sedimentation rate) were encountered. However, it should be noted that many authors did not discuss whether they opted to administer preoperative antibiotics, and this point should be treated with caution.

Most cases either placed low emphasis on or did not document the need for follow-ups. Nevertheless, we believe regular follow-up visits are important, especially during the first few years after resection. In cases where lesions have not been fully resected, attentive follow-ups are absolutely indicated.

Due to the rarity of cases, most CNMs are described in literature as case reports, and prospective studies are almost impossible to manage in these patients. In most cases, the prognosis is good. It is important to refer and manage these cases in tertiary centers and to involve MDTs in decision-making processes.

This is the first study to conduct a systematic literature review on this topic. In it, we have recommended an approach for managing newborns with CNMs based on our experience and updated literature review (Figure 4).

## Abbreviations

CNM: congenital nasopharyngeal mass
CT: computed tomography
EXIT: *ex-utero* intrapartum treatment
MDT: multidisciplinary team
MRI: magnetic resonance imaging
NICU: neonatal intensive care unit
